# Preimplantation Genetic Testing for Families at Risk of Haemophilia: Ten‐Year Single‐Centre Experience

**DOI:** 10.1111/hae.70358

**Published:** 2026-07-08

**Authors:** Mimosa Mortarino, Isabella Garagiola, Valeria Nicotra, Alessandra Riccaboni, Cristina Guarneri, Edgardo Somigliana, Flora Peyvandi

**Affiliations:** ^1^ Angelo Bianchi Bonomi Hemophilia and Thrombosis Center Fondazione IRCCS Ca’ Granda Ospedale Maggiore Policlinico and Fondazione Luigi Villa Milan Italy; ^2^ Medical Genetics Unit Fondazione IRCCS Ca' Granda Ospedale Maggiore Policlinico Milan Italy; ^3^ Infertility Unit Fondazione IRCCS Ca' Granda Ospedale Maggiore Policlinico Milan Italy; ^4^ Department of Pathophysiology and Transplantation Università Degli Studi di Milano Milan Italy

**Keywords:** carriers, hemophilia, pre‐implantation genetic testing, STR markers

## Abstract

**Introduction:**

Preimplantation genetic testing for monogenic diseases (PGT‐M) is a reproductive option for couples at high risk of transmitting inherited disorders. We report a ten‐year single‐centre PGT‐M experience in families at risk of hemophilia.

**Methods:**

A pre‐clinical PGT‐M work‐up was performed for couples in which the female was a carrier of hemophilia A or B, following multidisciplinary counselling. Intracytoplasmic sperm injection (ICSI) was used to generate embryos. Blastocysts on days 5–7 were biopsied for genetic testing and cryopreserved by vitrification. Short tandem repeat markers were used for linkage analysis, alone or in combination with the pathogenic variant using a multiplex PCR. Embryos with conclusive results were transferred one at a time.

**Results:**

23 couples contacted the center seeking information on the PGT‐M program, of which 19 underwent multidisciplinary counselling and pre‐clinical PGT work‐up. After ICSI, 69 embryos were biopsied and genetically characterized. A conclusive diagnosis was achieved for 58 embryos (84%): 42 unaffected embryos were suitable for transfer of whom 26 were transferred, resulting in 13 pregnancies (50% implantation rate per transfer), leading to eight live births, four miscarriages and one therapeutic termination. Prenatal diagnosis, performed in three cases, confirmed PGT‐M results. Amplification failure occurred in nine cases, eight of which were successfully re‐biopsied. Allele drop‐out occurred in two embryos and recombination in one.

**Conclusions:**

Our experience shows that PGT‐M is a valid and reliable option for couples at risk of transmitting severe genetic diseases, enabling prevention of affected pregnancies and reducing the emotional burden linked to therapeutic abortion.

## 1 Introduction

Preimplantation genetic testing for monogenic disorders (PGT‐M) represents a transformative advancement in reproductive medicine, enabling the avoidance of transmitting hereditary diseases through early genetic analysis. This approach allows couples at risk of transmitting a genetic disorder to begin a pregnancy with a disease‐free embryo, thereby avoiding invasive prenatal diagnosis and the potential interruption of the pregnancy in case of an affected male fetus, representing an option that is often more acceptable to most couples.

According to data from the European Society of Human Reproduction and Embryology (ESHRE) PGT Consortium, 1,388 PGT‐M analyses were conducted in 2018, with a mean female age of 32.9 years [1]. Nearly two‐thirds (64%) of these analyses were performed for autosomal dominant diseases, whereas autosomal recessive and X‐linked conditions accounted for 19% and 18% of cases, respectively [1].

A clinical application of PGT‐M is hemophilia, an X‐linked recessive bleeding disorder caused by a deficiency or dysfunction of coagulation factor VIII (FVIII) or factor IX (FIX). Haemophilia primarily affects males, leading to spontaneous bleeding episodes and prolonged bleeding following injury or surgery, whereas female carriers are usually asymptomatic, even though they may present variable bleeding symptoms [2, 3]. The significant morbidity associated with severe hemophilia A has made prenatal diagnosis and PGT‐M an attractive reproductive option for carrier females and their partners wishing to avoid having an affected child [4, 5].

PGT‐M is a well‐established diagnostic procedure that may involve the biopsy of different cell types, including polar body cells or embryonic cells at various stages of development, either blastomeres at the cleavage stage or trophectoderm cells at the blastocyst stage, allowing the detection of monogenic disorders before embryo transfer [6].

Historically, sex selection was the first reproductive strategy for X‐linked recessive disorders such as hemophilia, allowing only female embryos to be transferred [7]. Over time, multiplex PCR protocols using polymorphic short tandem repeat (STR) markers were optimized to detect the disease associated haplotypes along with the specific pathogenic gene variant in combination with sex‐linked markers on the X and Y chromosomes for sex determination [8]. Single‐cell analysis using multiplex PCR has long been the standard molecular approach for detecting monogenic disorders, typically applied after day‐3 blastomere biopsy [9]. In contrast, over the past decade, the introduction of blastocyst‐stage trophectoderm biopsy along with genome‐wide genetic platforms has largely replaced this previous strategy. These technologies enable simultaneous genotyping and chromosomal copy‐number assessment, allowing concurrent PGT‐M and PGT‐A for aneuploidy analyses [10, 11]. The first baby born free of hemophilia following PGT was reported in Colombia in 1994 [12]. Over the past three decades, PGT‐M programs have been successfully implemented in numerous medical centres worldwide, providing an alternative to prenatal diagnosis and possible pregnancy termination for hemophilia carrier couples.

In the present study, we report the ten‐year experience (2015–2025) of the Angelo Bianchi Bonomi Hemophilia and Thrombosis Center, in collaboration with the Infertility and Medical Genetics Units, Fondazione IRCCS Ca' Granda Ospedale Maggiore Policlinico, Milan, Italy, following the 2015 ruling of the Italian Constitutional Court (Decision No. 96/2015), which legalised embryo genetic analysis for couples at high genetic risk.

## 2 Materials and Methods

### 2.1 Subjects and Counselling

Couples in which the female was a genetically diagnosed carrier of hemophilia A or B were prospectively enrolled and underwent multidisciplinary genetic counselling which is the first step of the PGT program. Each couple received all the information regarding the assisted reproductive technology (ART) and specific genetic counselling addressing all aspects of PGT‐M. Counselling was provided by a multidisciplinary team including a gynaecologist, a geneticist, and a psychologist.

The following documentation was required for each couple:

- The genetic report relating to the monogenic disorder, karyotype analysis, or any other relevant data relating to the couple and/or the family and the proband;
- The female partner reproductive history, gynaecological evaluation, transvaginal ultrasound and additional fertility investigations, if needed;
- The male partner reproductive history, semen analysis, and andrological evaluation, if needed.

Couples at risk of hemophilia received disease‐specific counselling from a hematologist and a geneticist at the Hemophilia Center. Female carriers underwent biochemical characterization, including coagulation factor measurement, evaluation of bleeding history, and collection of family genetic information. Psychological support was offered throughout the entire PGT‐M process.

### 2.2 Preclinical PGT‐M Work‐Up

Prior to clinical application, each case underwent a preclinical validation work‐up using peripheral blood DNA and buccal cell DNA from both parents and from an affected family member. A written informed consent was obtained from all couples prior to testing. A panel of intragenic and/or extragenic polymorphic STR markers, located within 1 Mb (∼1 cM) of the *F8* or *F9* gene to minimize recombination risk (on average, loci 1 cM apart are expected to show 1% recombination) was selected (Table 1). STRs were co‐amplified with the pathogenic variant by multiplex PCR, except for cases involving *F8* intron 22 or intron 1 inversions, or large deletions/duplications, in which only STRs were used. One primer of each STR pair was fluorescently labelled for fragment analysis and peak quantification by capillary electrophoresis (3130 Genetic Analyzer, Applied Biosystems) [13]. The preclinical work‐up assessed both STR informativity and the feasibility of direct detection of the pathogenic gene variant. STR genotyping was performed on familial DNA to identify informative markers and to identify the high‐risk haplotype segregating along with the pathogenic variant. Based on these results, either an indirect haplotype‐based approach or a direct approach combining haplotype analysis with gene variant detection was established.

**TABLE 1:** Information on STR intragenic and extragenic markers for the F8 and F9 genes. F8 and F9 gene locations on chromosome X according to the UCSC Genome Browser (hg19, corresponding to GRCh37) are: F8, chrX:154,064,067–154,250,998; F9, chrX:138,612,898–138,645,618.

	STR name	Chromosome X localization (GRCh37/hg19)	hg19 STS marker
3′ extragenic markers of F8	DXS8061	152021508–152021832	AFMB311YG1
	DXS15	152612043–152612355	DXS15
	DXS9901	152773987–152774993	CHLC.UTR_05745_U11686
	DXS8087	152894326–152894670	AFM312ZB9
	DXS1073	153828848–153829168	AFM276XH9
*Intragenic markers of F8*	*F8IVS24*	*154078310*–*154078520*	—
	*F8IVS22*	*154103951*–*154104170*	—
	*F8 int21*	*154124741*–*154124890*	—
	*F8IVS13*	*154164230*–*154164376*	—
	*F8 int9.2*	*154191117+154191282*	—
5′ extragenic markers of F8	HEMA 154507.3	154853972–154854271	—
	DXS1108	154861849–154862012	DXS1108
5′ extragenic markers of F9	DXS1211	138305230–138305574	AFM276VF9
	DXS1192	138368034–138368235	AFM196XA1
	DXS102	138383368–138383514	DXS102
3′ extragenic markers of F9	DXS1232	139280104–139280361	AFMA123YB5
	DXS8013	139486121–139486376	AFM144YD2
	DXS984	139631728–139631928	AFM105XC5

*Note*: Chromosome X localization is based on the UCSC Genome Browser (hg19), corresponding to the Genome Reference Consortium human assembly GRCh37.

Abbreviations: STR, short tandem repeat; STS, sequence‐tagged site.

Couple were eligible to proceed with the PGT‐M cycle only if the preclinical work‐up confirmed the presence of a sufficient number of informative markers, at least two located at the 5′ end and two at the 3′ end of the gene under investigation, according to ESHRE guidelines [14]. The accuracy of the protocol was required to exceed 99%, with an estimated error rate of approximately 1%.

### 2.3 Ovarian Stimulation, in Vitro Fertilization (IVF) and Embryo Biopsy

Female carriers underwent ovarian stimulation to retrieve multiple oocytes according to standard clinical protocols [15, 16]. ICSI was performed to fertilize the oocytes and generate embryos in vitro, thereby minimizing the risk of sperm DNA contamination. Embryos were maintained in culture until the blastocyst stage. Genetic analysis at the blastocyst stage (5th–7th day of in vitro development) was performed by biopsy of 5–10 cells from the trophectoderm. The use of laser was the preferred method for biopsy. Following biopsy, blastocysts were cryopreserved by vitrification.

Before biopsy, blastocyst quality was assessed using the Gardner blastocyst grading system, as recommended by the ESHRE/ALPHA Istanbul Consensus Update Working Group [17]. This system evaluates the developmental stage of the blastocyst and the morphology of both the inner cell mass and the trophectoderm. After PGT analysis unaffected embryos with the best morphology were selected for transfer irrespective of their sex or carrier status.

### 2.4 Genetic Analysis and Embryo Transfer

After alkaline lysis of embryo biopsied cells, informative STR markers identified during the preclinical PGT‐M work‐up were used for linkage analysis, either alone or in combination with the family‐specific pathogenic variant, using multiplex PCR (3500 Genetic Analyzer, Applied Biosystems). Additionally, three sex‐linked markers (AMELX/Y, SRY, and DYS14) were used for embryo sex determination. Following genetic diagnosis, couples received post‐test counselling to communicate the results of embryo analysis.

Embryos with a conclusive diagnosis and deemed suitable for transfer were transferred one at a time, while the other embryos remained cryopreserved. Frozen embryo transfers were preferentially scheduled within a natural menstrual cycle [18].

### 2.5 Coagulation Assays

FVIII and FIX coagulant activity (FVIII:C and FIX:C) were measured using one‐stage clotting assay as previously described on blood samples collected in a 3.2% buffered citrate solution [19].

## 3 Results

A total of 23 couples contacted the Hemophilia and Infertility Centers at Fondazione IRCCS Ca' Granda Ospedale Maggiore Policlinico, Milan, Italy, to undergo PGT‐M procedure (Table 2). Three couples requested information about the program, but did not proceed beyond preliminary counselling, and one couple attended the multidisciplinary consultation and subsequently discontinued the program (Table 2). The remaining nineteen couples completed initial multidisciplinary counselling, signed the informed consent for testing, and provided the biological samples needed for subsequent genetic analysis. The preclinical PGT‐M work‐up was completed for all couples; however, two couples discontinued the process, even though the PGT‐M work‐up was informative. In two other cases, STR polymorphic markers analysed were non informative, and therefore the embryo analysis was not feasible (Table 2).

**TABLE 2:** Characteristics of couples undergoing multidisciplinary counselling and data from analysed embryo biopsies.

	HA	HB	TOT
Couples (Total)	18	5	23
*Couples requiring information only*	3	1	4
Couples counseled (*n*)	15	4	19
Couples discontinued PGT program (*n*)	2	0	2
Couples with negative preclinical PGT work‐up (*n*)	2	0	2
Couples underwent PGT cycles (*n*)	11	4	15
Embryos byopsied and analyzed	47	22	69
Embryos diagnosed *n* (%)	41 (87%)	17 (77%)	58 (84%)
*Trasferable embrios*	30	12	42
*Untrasferable embrios*	10	5	15
*Embryos with allele recombination*	1	0	1
Embryos no diagnosed *n* ^a^	4	5	9
*Embryos rebiopsied*	4	4	8^c^
*Embryos diagnosed n (%)*	4 (100%)	4 (100%)	8 (100%)
Inconclusive analysis^b^	2	0	2
Embryo transfer	19	7	26
Positive hCG test	9	4	13
Implantation rate (per ET) (%)	9	4	13 (50%)
Clinical pregnancies	7	1	8
*Live births*	6	2	8
*Female*	5	0	5
*Male*	1	2	3
*Miscarriages*	2	2	4
*Termination of pregnancy*	0	1	1
Prenatal diagnosis	1	2	3
Embryos still remain cryopreserved	28	14	42

^a^ Amplification failed for all markers and for specific gene variant.

^b^ Allele‐drop out.

^c^ One embryo did not survive thawing for second biopsy.

Fifteen couples, eleven with hemophilia A and four with hemophilia B, underwent PGT‐M cycles. The mean maternal age of carrier women, who underwent hormonal stimulation and oocyte pickup, was 34.6 ± 3.8 years (range, 27 to 41), as reported in Table 3. To reduce the risk of bleeding episodes during oocyte pickup, specific hemostatic management was applied according to each carrier's coagulation deficiency to ensure factor levels >50 IU/dL. Four female carriers of hemophilia A were found to have reduced FVIII activity levels, with a mean of 44.0 IU/dL (range: 39–50 IU/dL). These carriers were treated with desmopressin (DDAVP). One carrier had a FVIII level of 60 IU/dL measured by one‐stage assay and 40 IU/dL measured by chromogenic assay. Notably, this carrier had a history of severe bleeding (Table 2: couple PGT.17), and prophylactic tranexamic acid was consequently recommended. Two carriers of hemophilia B had FIX activity levels of 33 IU/dL and 40 IU/dL, respectively and were managed with recombinant FIX concentrates (Benefix) (Table 2).

**TABLE 3:** Demographic, genetic and treatment‐related factors in carriers undergoing PGT.

Couple ID	Maternal age	Disease	Relatedness with hemophilic patients	F8/F9 mutation (HGVS nomenclature)	^a^FVIII or ^b^FIX coagulant level (IU/dL)	Treatment before pick‐up	Number of cycles
PGT.1	41	HA	daughter	c.6429+16424_6430−16424inv	111	No	1
PGT.2	39	HA	cousin	c.143+?_144−?inv	53	No	1
PGT.3	35	HA	mother	p.Arg2326Leu (c.6977G>T)	48	Emosint‐DDAVP (0,3 mcg/kg)	1
PGT.4	27	HA	daughter and mother	c.6429+16424_6430−16424inv	67	No	1
PGT.5	35	HA	daughter	p.Trp604stop (c.1812G>A)	50	Emosint‐DDAVP (0,3 mcg/kg)	1
PGT.8	35	HA	mother	c.6429+16424_6430−16424inv	83	No	0
PGT.13	31	HA	mother	c.6429+16424_6430−16424inv	101	No	0
PGT.14	38	HA	daughter	c.6429+16424_6430−16424inv	69	No	6
PGT.15	36	HA	aunt	p.Arg2326Leu (c.6977G>T)	67	No	5
PGT.16	38	HA	daughter	p.Arg2182Pro (c.6545G>C)	55	No	0
PGT.17	35	HA	mother	c.6429+16424_6430−16424inv	60	^c^Prophylaxis with tranexamic acid	4
PGT.18	33	HA	mother	c.6429+16424_6430−16424inv	86	No	3
PGT.19	27	HA	mother	p.Ala2058Pro (c.6172G>C)	39	Emosint‐DDAVP (0,3 mcg/kg)	1
PGT.20	32	HA	daughter	p.Arg490_Pro491dup (c.1467_1472dupCAGACC)	39	Emosint‐DDAVP (0,3 mcg/kg)	1
PGT.23	36	HA	mother	c.6429+16424_6430−16424inv	32	^d^NA	0
PGT.7	33	HB	daughter	p.Arg384stop (c.1150C>T)	40	Benefix (4000U)	1
PGT.12	39	HB	daughter	c.88+5_88+8delCTTT	86	No	3
PGT.21	36	HB	daughter	p.Gly236Asp (c.707G>A)	33	Benefix (2000U)	5
PGT.22	33	HB	cousin	p.Arg43Trp (c.127C>T)	63	No	7

Abbreviation: NA, not applicable.

^a^ Normal range 50–140 IU/dL.

^b^ Normal range 70–120 IU/dL.

^c^ The treatment was also determined on the basis of FVIII activity data obtained from the chromogenic assay.

^d^ Treatment will be defined before oocytes pick‐up.

Of the 15 couples enrolled in the PGT‐M program, eight underwent a single PGT cycle, whereas seven couples underwent multiple cycles, ranging from three to seven (Table 3).

A total of 69 embryos (47 from hemophilia A couples and 22 from hemophilia B couples), obtained via ICSI, reached the blastocyst stage and were biopsied (Table 2). Genetic analysis was carried out on the sampled trophectoderm cells. In nine cases, PGT‐M was performed exclusively by haplotype analysis, as direct amplification of the intron 22 and intron 1 inversions was not technically feasible in DNA samples derived from a limited number of trophectoderm cells.

A conclusive diagnostic result was achieved in 58 embryos (84%, 58/69) of which 72%, (42/58) were genetically transferable. Genetic analysis in a female embryo revealed allele recombination involving three STR markers (DXS8377, DXS8061, and DXS9901) located upstream (5′) of the F8 gene (Table 1). After genetic counselling, the couple decided to transfer the single embryo anyway, albeit recombinant, but a miscarriage occurred shortly thereafter.

Nine embryos did not receive a conclusive diagnosis due to complete PCR amplification failure of all STR markers. Eight of these embryos underwent re‐biopsy and were successfully genotyped. Inconclusive results were obtained for two embryos due to a partial allele drop‐out affecting STR markers or amplification failure of the pathogenic variant.

Transfer of 26 cryopreserved embryos resulted in a positive human chorionic gonadotropin (hCG) in 13 pregnancies with an implantation rate of 50% (13/26) per embryo transfer (ET). Pregnancies resulted in 8 live births (7 females and 1 male) (live birth rate of 31%), 4 miscarriages and 1 therapeutic abortion. All live births were free of disease. Forty‐two embryos still remain cryopreserved, twenty‐four of which, are transferable. Three couples underwent prenatal diagnosis and results were consistent with PGT genetic analysis.

## 4 Discussion

Our ten‐year experience with PGT‐M confirms that this technique represents a reliable reproductive option for couples at risk of transmitting X‐linked disorders such as hemophilia. The couples opted for PGT‐M to prevent the transmission of the disease [5] and this choice was consistent with recent studies showing that couples primarily aim to have a genetically related child free from a specific disorder avoiding termination of pregnancy [20, 21].

Multidisciplinary counselling was offered to couples at all stages of PGT‐M procedure, and all medical specialists were available to address any needs or doubts throughout each step of the process. Appropriate psychological support was offered to couples throughout the entire route, including cases of unsuccessful cycles. Such support was particularly recommended for couples with a history of traumatic experiences due to repeated reproductive failures/pregnancy interruption or evident stress related to reproductive issues; for those who have previously had a child with hemophilia; or for couples who specifically requested psychological counselling.

In our cases, preclinical work‐up was generally performed by testing STR markers flanking the pathogenic gene variant to identify the segregating at‐risk haplotype. In only two hemophilia A cases, the preclinical work‐up failed to identify sufficient informative STR markers.

Newer approaches, such as karyomapping using SNP arrays, could be useful. This technique uses SNP‐array technology to analyse thousands of SNPs across the genome and define an individual's genotype, although each SNP is biallelic and therefore less informative than highly polymorphic STRs, a set of four SNP markers is necessary to establish the genotypes of the parents and the index case/proband [10].

In line with previous reports, ICSI was the fertilisation method preferentially used in all hemophilia couples. Blastocyst biopsy was applied in all cases, despite the data reported by the ESHRE PGT consortium analysis in 2018 [1], in which cleavage stage biopsy remains the most common approach in PGT‐M (65%) while blastocyst biopsy is applied in approximately 33% of cases [1]. This procedure was adopted in our center because it yields a greater amount of DNA, allowing for a more accurate genetic diagnosis. An additional advantage is that the biopsy involves trophectoderm cells, which do not contribute directly to fetal development. Finally, several studies have demonstrated that embryos vitrified at the blastocyst stage exhibit higher post‐thaw survival rates compared with those vitrified at the cleavage stage [22]. Furthermore, cryopreservation guarantees more time to carry out the genetic analysis.

The efficacy of our diagnostic testing was satisfactory, 84% (58/69) of embryos analysed gave a genetic result, and 74% (43/58) were genetically transferable. Embryos were transferred individually, following international guidelines. In accordance with international guidelines, the embryos were transferred individually, which reduces miscarriage risk and minimizes twin pregnancies and associated complications.

All biopsies were analysed using multiplex PCR of STR markers co‐amplified with the pathogenic variant, consistent with literature indicating PCR as the first‐line method in 85% of cases, despite the recent implementation of whole genome amplification (WGA) and NGS technologies [1].

In 8 cases with inconclusive results, a second biopsy was performed, resulting in a conclusive diagnosis in all cases. Three re‐biopsied embryos were transferred without achieving a pregnancy, in line with recently published data reporting a low pregnancy rate (∼30%) after re‐biopsied blastocyst transfer [23].

Overall, the live birth rate was 31% (8/26), consistent with recent reports [1].

Currently, no international consensus exists regarding the use of prenatal diagnosis in pregnancies achieved following the transfer of embryos tested by PGT‐M. During both pre‐ and post‐PGT‐M counselling, medical specialists in our center suggested prenatal diagnosis through either invasive procedures or non‐invasive prenatal testing. In our couple, an invasive prenatal diagnosis by chorionic villus sampling was performed in three cases, confirming in all of them the results of PGT‐M.

## 5 Conclusions

Our ten‐year experience with PGT‐M confirms that this technique is a complex but robust and reliable reproductive strategy. Furthermore, it can inform couples about the health of their embryos before pregnancy begins, minimising the risk of transmitting severe genetic disorders, reducing the need for pregnancy termination in case of an affected fetus, and alleviating the emotional burden associated with such procedures.

## Author Contributions

Mimosa Mortarino performed the PGT‐M genetic analysis. Alessandra Riccaboni, Valeria Nicotra, and Edgardo Somigliana were responsible for counselling, ovarian stimulation, and embryo transfer. Cristina Guarneri performed in vitro fertilization and embryo biopsy. Isabella Garagiola, Mimosa Mortarino and Flora Peyvandi designed, collected and analysed the data and drafted the manuscript. All authors contributed to data interpretation, critically revised the manuscript, and approved the final version.

## Funding

The authors have nothing to report.

## Conflicts of Interest

Flora peyvandi: Participation on a Data Safety Monitoring Board or Advisory Board: BioMarin, Roche, Sobi, Sanofi, Pfizer, CSL Behring. Honoraria for lectures, presentations, speakers’ bureaus, manuscript writing or educational events: Sanofi, Takeda, Sobi, BioMarin. Edgardo somigliana received honoraria for presentations at international meetings from Ipsen, Gedeon‐Richter and IBSA. He also did two consultations for Ferring, and he is handling a grant of research from IBSA. Finally, he is participating to an observational study supported by Merck‐Serono. Mimosa mortarino, Isabella garagiola, Alessandra riccaboni, Cristina guarneri: have no conflicts of interest.

## Data Availability

The data that support the findings of this study are available on request from the corresponding author. The data are not publicly available due to privacy or ethical restrictions.
